# Determination of the optimum definition of growth evaluation for indeterminate pulmonary nodules detected in lung cancer screening

**DOI:** 10.1371/journal.pone.0274583

**Published:** 2022-09-15

**Authors:** Jong Hyuk Lee, Eui Jin Hwang, Woo Hyeon Lim, Jin Mo Goo

**Affiliations:** 1 Department of Radiology, Seoul National University Hospital, Seoul, Korea; 2 Department of Radiology, Seoul National University College of Medicine, Seoul, Korea; 3 Institute of Radiation Medicine, Seoul National University Medical Research Center, Seoul, Korea; 4 Cancer Research Institute, Seoul National University, Seoul, Korea; Emory Healthcare, UNITED STATES

## Abstract

**Objective:**

To determine the optimum definition of growth for indeterminate pulmonary nodules detected in lung cancer screening.

**Materials and methods:**

Individuals with indeterminate nodules as defined by volume of 50–500 mm^3^ (solid nodules) and solid component volume of 50–500 mm^3^ or average diameter of non-solid component ≥8 mm (part-solid nodules) on baseline lung cancer screening low-dose chest CT (LDCT) were included. The average diameters and volumes of the nodules were measured on baseline and follow-up LDCTs with semi-automated segmentation. Sensitivities and specificities for lung cancer diagnosis of nodule growth defined by a) percentage volume growth ≥25% (defined in the NELSON study); b) absolute diameter growth >1.5 mm (defined in the Lung-RADS version 1.1); and c) subjective decision by a radiologist were evaluated. Sensitivities and specificities of diagnostic referral based on various thresholds of volume doubling time (VDT) were also evaluated.

**Results:**

Altogether, 115 nodules (one nodule per individual; 93 solid and 22 part-solid nodules; 105 men; median age, 68 years) were evaluated (median follow-up interval: 201 days; interquartile range: 127–371 days). Percentage volume growth ≥25% exhibited higher sensitivity but lower specificity than those of diametrical measurement compared to absolute diameter growth >1.5 mm (sensitivity, 69.2% vs. 42.3%, *p* = 0.023; specificity, 82.0% vs. 96.6%, *p = 0*.*002*). The radiologist had an equivalent sensitivity (53.9%; *p* = 0.289) but higher specificity (98.9%; *p* = 0.002) compared to those of volume growth, but did not differ from those of diameter growth (*p*>0.05 both in sensitivity and specificity). Compared to the VDT threshold of 600 days (sensitivity, 61.5%; specificity, 87.6%), VDT thresholds ≤200 and ≤300 days exhibited significantly lower sensitivity (30.8%, *p* = 0.013) and higher specificity (94.4%, *p* = 0.041), respectively.

**Conclusion:**

Growth evaluation of screening-detected indeterminate nodules with volumetric measurement exhibited higher sensitivity but lower specificity compared to diametric measurements.

## Introduction

With cumulative evidence of lung cancer mortality reduction, lung cancer screening with low-dose chest CT (LDCT) for a high-risk population is recommended [1–4]. Resultantly, a nationwide screening program has been implemented in various countries across the world [1–4]. Defining the positive screening results requiring additional LDCT or diagnostic evaluation (e.g., biopsy, surgery) is paramount to lung cancer screening [3, 5–7]. With an inclusive threshold, a considerable number of false-positive results lead to increased medical resource usage without substantial benefits, as well as additional radiation exposure, complications from unnecessary invasive procedures, and potential psychosocial consequences for participants [3, 5–7]. Conversely, a conservative threshold could delay or miss the detection of lung cancer [3, 5–7].

Positive baseline screening LDCT results are defined based on the size and consistency of pulmonary nodules, and participants with indeterminate pulmonary nodules underwent follow-up LDCTs [5–7]. Meanwhile, in the follow-up LDCTs, the presence of nodule growth and growth rate are key components for defining positive results that require invasive diagnostic procedures [4, 8, 9]. Conventionally, growth assessment of a pulmonary nodule is based on uni- or bi-dimensional diametrical measurement [10–12], and the lung CT screen reporting and data system (Lung-RADS) from the American College of Radiology defines nodule growth as an absolute increase in average diameter >1.5 mm [13]. Meanwhile, volumetric measurement is expected to detect nodule growth more sensitively and reduce inter- and intra-reader variability [12, 14]. In the Dutch-Belgian lung cancer screening trial (NELSON), the growth of nodules was defined as a relative increase in nodule volume greater than 25% and a volume doubling time (VDT) <400 days indicated positive results necessitating a diagnostic referral [4, 8, 9].

However, there is limited research on the definition of nodule growth and the growth rate is optimal for identifying lung cancer among indeterminate-sized pulmonary nodules, defined as volume of 50–500 mm^3^ (solid nodules) and solid component volume of 50–500 mm^3^ or average diameter of non-solid component ≥8 mm (part-solid nodules), detected in baseline screening LDCTs. Therefore, we aimed to evaluate the diagnostic accuracy of different criteria (i.e., diametrical measurement, volumetric measurement, and subjective decision by a radiologist) for growth and diagnostic referral of indeterminate pulmonary nodules for lung cancer in lung cancer screening.

## Materials and methods

The Institutional Review Board of Seoul National University Hospital approved this study and waived the requirement for informed consent from the patients.

### Study population

**Fig 1:**
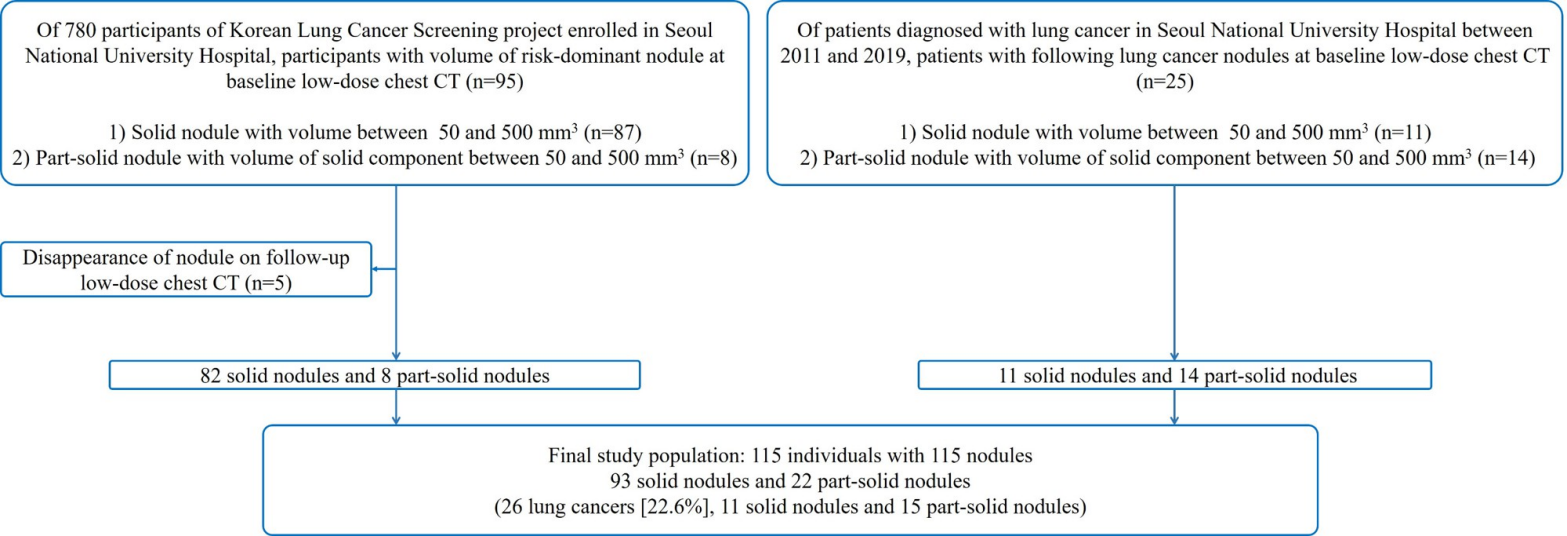
Flowchart of the study population.

We enrolled the study population from two consecutive cohorts: (a) participants of The Korean Lung Cancer Screening (K-LUCAS) project enrolled in our institution between 2017 and 2018 [15, 16]; and (b) subjects who underwent screening LDCTs for a health check-up at our institution and were consequently diagnosed with lung cancer between 2011 and 2019. The common inclusion criteria for the two cohorts were: (a) Individuals with indeterminate baseline LDCT results defined by the criteria of NELSON (i.e., solid nodules with a volume 50–500 mm^3^; part-solid nodules with a volume of solid component 50–500 mm^3^ or average diameter of non-solid component ≥8 mm; non-solid nodule with average diameter ≥8 mm) [4, 9]; (b) individuals with follow-up LDCT for nodules detected on baseline LDCT; and (c) individuals with solid or part-solid nodules, not non-solid nodule. Individuals with nodules that disappeared in the follow-up LDCT in the K-LUCAS project were excluded (Fig 1). In this study, we defined lung cancers as pathologically proven pulmonary nodules and otherwise regarded as benign nodules.

For individuals with two or more nodules on baseline LDCTs, one dominant nodule was selected with the following criteria because the most suspicious nodule guides the patients’ management strategy [11]: (a) the nodule with the largest volume (volume of solid component for part-solid nodules) was selected and (b) solid nodules were accorded priority over part-solid nodules.

Consequently, we included 115 indeterminate nodules from 115 individuals in this study.

### LDCT examination

CT examinations were performed using one of the nine different scanners from four manufacturers (Brilliance 64, Ingenuity, iCT, IQon [Phillips Medical Systems, Best, Netherlands]; Somatom Sensation 16, Somatom Definition, Sonatin Force [Siemens Medical Solutions, Forchheim, Germany]; Aquilion One [Canon Medical Systems, Otawara, Japan]; Discovery CT750HD [GE Medical Systems, Waukesha, United States]). Common acquisition and reconstruction protocols were: (a) Image acquisition in a single breath-hold at full inspiration with supine position; (b) transverse CT image reconstruction with a high-frequency algorithm with slice thickness <1.5 mm without an inter-slice gap. The same CT scanner was used for baseline and follow-up LDCTs in 26.1% (30 of 115) of individuals, while different CT scanners were used in the remaining 73.9%.

### Nodule measurement

**Fig 2:**
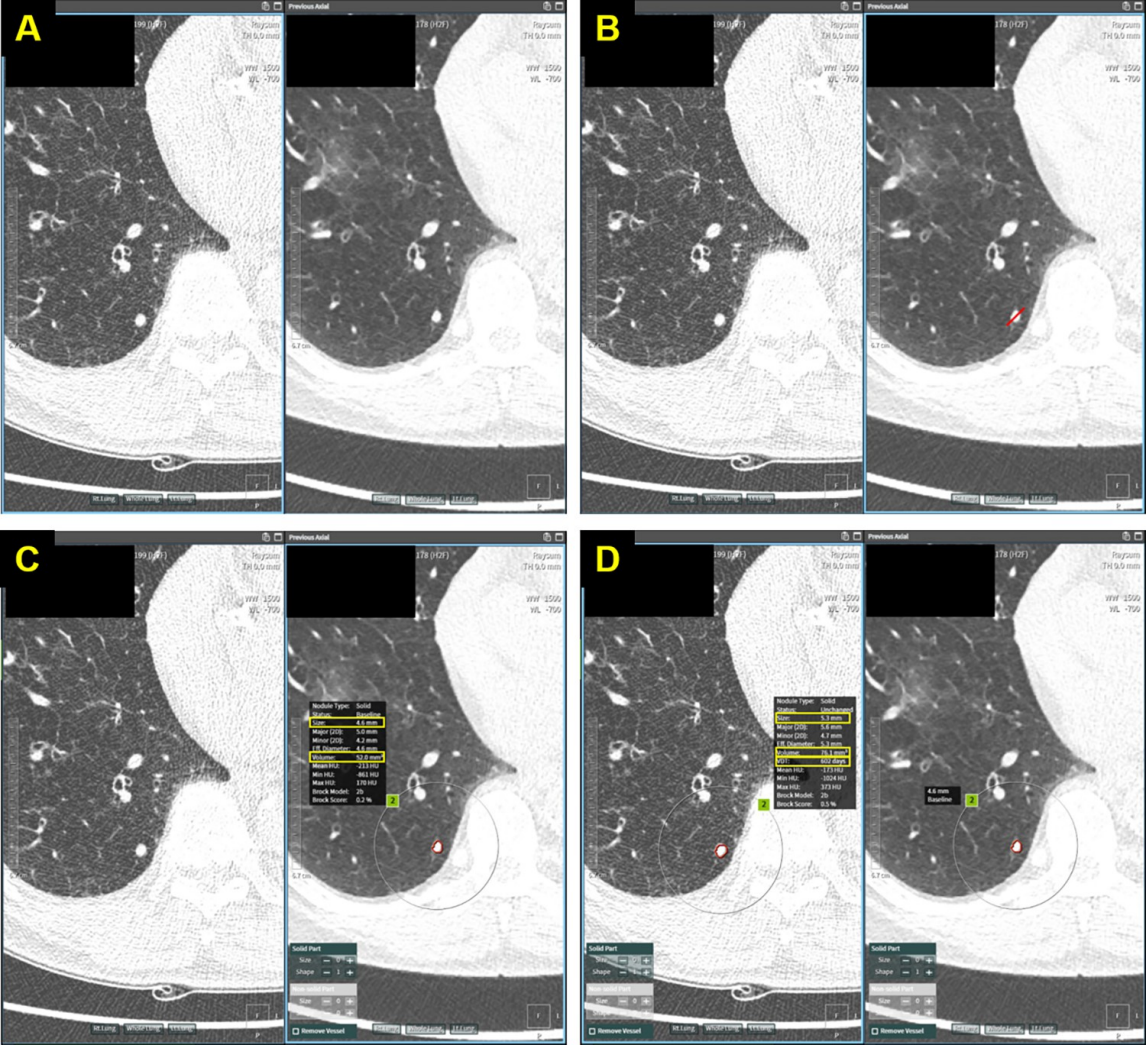
Semi-automated measurement of pulmonary nodules. (A) Baseline low dose chest CT (LDCT, right side) and follow-up LDCT (left side) show a solid nodule at right lower lobe of the lung. (B) Semi-automated segmentation and measurement of the nodule can be initiated by drawing a line on the nodule. (C) The software can visualize the result of semi-automated segmentation as well as the result of measurement. On the baseline LDCT, average diameter and volume of the nodule were 4.6 mm and 52.0 mm^3^, respectively. (D) On the follow-up LDCT, the average diameter and volume of the nodule were 5.3 mm and 76.1 mm^3^, respectively. Consequently, absolute diameter growth and percentage volume growth of the nodule were 0.7 mm and 31.7% respectively, and the volume doubling time was 602 days.

To evaluate nodule size, a thoracic radiologist (E.J.H., 11-year experience in chest CT interpretation) measured the indeterminate pulmonary nodules on the baseline and follow-up LDCTs using a commercial software (A-view LungScreen, Coreline Soft). By designating the target nodule by the user, the software automatically segmented the boundary of the nodule (separate segmentation of ground-glass component and solid component for part-solid nodules). If segmentation by the software is not judged to be appropriate by the user, users can adjust the segmentation manually. Subsequently, the software provided the maximum average diameter measured on the transverse plane (average diameter, hereafter) and volume of the target nodule based on the segmentation results (Fig 2). Indeed, this software was implemented during the first year of The K-LUCAS project, and attending thoracic radiologists in the participating institutions read lung cancer screening LDCT using this software [16–18].

To evaluate inter-reader agreement of the semi-automated nodule measurement, 14 randomly sampled nodules (solid nodules, n = 8; part-solid nodules, n = 6) were independently measured by another thoracic radiologist (J.H.L, 9-year experience in chest CT interpretation) using the same software.

### Evaluation of nodule growth

To evaluate nodule growth, the following metrics were evaluated for each nodule based on the semi-automated measurement (separate evaluation of the whole nodule including ground-glass component and solid component for part-solid nodules):

$$\begin{array}{l} (a)\;Percentage\;volume\;growth\;(\%)=\\ \frac{Volume\;on\;follow‐up\;LDCT\;({mm}^{3})-Volume\;on\;baseline\;LDCT({mm}^{3})}{Volume\;on\;baseline\;LDCT\;({mm}^{3})}\times 100 \end{array}$$

$$\begin{array}{l} (b)\;Absolute\;diameter\;growth\;(mm)=\\ Average\;diameter\;on\;follow‐up\;LDCT\;(mm)-Average\;diameter\;on\;Baseline\;LDCT\;(mm) \end{array}$$

$$\begin{array}{l} (c)\;Volume\;doubling\;time\;(VDT,days)=\\ \frac{\ln\;2\times (Date\;of\;follow‐up\;LDCT-Date\;of\;baseline\;LDCT)}{\ln\frac{Volume\;on\;follow‐up\;LDCT\;({mm}^{3})}{Volume\;on\;baseline\;LDCT\;({mm}^{3})}} \end{array}$$

VDTs were evaluated in nodules that exhibited a percentage volume growth ≥25%, as suggested by the NELSON and European position statement [4, 8, 9, 19].

For subjective evaluation of nodule growth, one thoracic radiologist (J.H.L.) and one general radiologist (W. H. L., 7 years of experience in chest CT interpretation) who was blinded to the diagnosis of lung cancer reviewed baseline and follow-up LDCTs and decided whether (a) there was any growth of the nodule and (b) diagnostic process other than follow-up LDCT is required (diagnostic referral, hereafter). For the subjective evaluation of nodule growth, measurement of nodules using an electronic caliper was allowed, but the semi-automated measurement results, including nodule volume, were not provided. Inter-reader agreement was evaluated between the interpretation of the two radiologists, while only interpretation by one radiologist (J.H.L.) was used for the performance evaluation.

To evaluate the diagnostic performance for lung cancer in different definitions of nodule growth, we evaluated three different definitions of nodule growth: (a) Percentage volume growth of ≥25% (as defined in the NELSON) [4, 8, 9]; (b) absolute diameter growth of >1.5 mm (per the Lung-RADS) [13]; and (c) any growth defined by the subjective evaluation of the radiologist. For part-solid nodules, growth of either the ground-glass or solid components was considered growth.

We evaluated the diagnostic performance of different thresholds of VDT (VDT of 600, 500, 400, 300, 200, and 100 days) and the radiologists’ subjective diagnostic referrals for diagnosing lung cancer.

### Statistical analysis

To evaluate the diagnostic performance for lung cancer of each growth metric (i.e., percentage volume growth, absolute diameter growth, and VDT), we performed receiver-operating characteristic curve analyses, and area under the receiver-operating characteristic curves (AUCs) were obtained. Sensitivity and specificity were obtained for diagnostic performance at specific thresholds of growth and diagnostic referral. Comparison of AUCs was performed using the method suggested by DeLong, while comparisons of sensitivities and specificities were performed using McNemar tests. Subgroup analyses were performed in the same manner, with nodules presenting as solid nodules on baseline screening CTs.

Inter-reader agreement for semi-automated lung nodule measurement was evaluated using the interclass correlation coefficient and Bland-Altman plots [20, 21], while inter-reader agreements for nodule growth and diagnostic referral by radiologists’ subjective interpretation were evaluated with percentage agreement and Cohen’s kappa coefficient [22].

All statistical analyses were performed using Medcalc version 20.009 (MedCalc Software Ltd) and R version 4.1.0 (R Project for Statistical Computing), and a *p*-value of <0.05 was considered to indicate statistical significance.

## Results

### Baseline characteristics

Of the 115 individuals included in the study (men, n = 115; women, n = 10; median age 68 years, interquartile range [IQR], 63–72 years), 46 were current smokers, 59 were former smokers, and 10 were non-smokers. In 112 individuals with pack-year information, the median smoking burden was 40 pack-years (IQR, 30–43 pack-years). The median time interval between baseline and follow-up LDCT was 201 days (IQR, 127–371 days). Of the 115 nodules, 93 were solid nodules, 22 were part-solid nodules, and 26 were lung cancers (22.6%, solid nodules, n = 11; part-solid nodules, n = 15). The average diameter and volume of the 115 nodules in the baseline and follow-up LDCT are tabulated in Table 1.

**Table 1:** Volume and average diameter of the 115 nodules measured in baseline and follow-up screening low-dose chest CT.

Criteria	Group	Subgroup	Baseline LDCT	Follow-up LDCT	Number of growing nodules*
Volume (mm^3^)	Total nodules (n = 115)	Total nodules (n = 115)	341.0mm^3^ ± 497.8 (51.7–3372.4mm^3^)	427.9mm^3^ ± 638.7 (22.8–3229.7mm^3^)	34 (29.6%)
		Solid nodules (n = 93)	167.5mm^3^ ± 122.3 (51.7–167.5mm^3^)	198.9mm^3^ ± 183.2 (22.8–894.3mm^3^)	22 (23.7%)
		Part-solid nodules (n = 22)	1074.6mm^3^ ± 763.4 (218.2–3372.4mm^3^)	1395.9mm^3^ ± 924.1 (279.5–3229.7mm^3^)	12 (54.5%)
		Solid component of part-solid nodules (n = 22)	104.1mm^3^ ± 109.4 (5.2–411.1mm^3^)	176.4mm^3^ ± 232.2 (3.3–1036.5mm^3^)	Not applicable
	Cancer nodule (n = 26)	Total cancer nodules (n = 26)	850.1mm^3^ ± 806.5 (52.1–3372.4mm^3^)	1203.3mm^3^ ± 935.6 (227.9–3229.7mm^3^)	18 (69.2%)
		Solid nodules (n = 11)	287.0mm^3^ ± 152.6 (52.1–488.8mm^3^)	549.4mm^3^ ± 239.5 (227.9–894.3mm^3^)	9 (81.8%)
		Part-solid nodules (n = 15)	1262.9mm^3^ ± 844.5 (218.2–3372.4mm^3^)	1682.9mm^3^ ± 969.4 (462.0–3229.7mm^3^)	9 (60%)
		Solid component of part-solid nodules (n = 15)	127.4mm^3^ ± 124.1 (6.8–411.1mm^3^)	224.3mm^3^ ± 268.4 (13.0–1036.5mm^3^)	Not applicable
	Benign nodule (n = 89)	Total benign nodules (n = 89)	192mm^3^ ± 193.2 (51.7–1153.8mm^3^)	201.4mm^3^ ± 224.2 (22.8–1302.9mm^3^)	14 (15.7%)
		Solid nodules (n = 82)	151.5mm^3^ ± 109.1 (51.7–496.1mm^3^)	151.9mm^3^ ± 110.1 (22.8–110.1mm^3^)	11 (13.4%)
		Part-solid nodules (n = 7)	670.9mm^3^ ± 311.0 (354.1–1153.8mm^3^)	780.7mm^3^ ± 385.1 (279.5–1302.9mm^3^)	3 (42.9%)
		Solid components of part-solid nodules (n = 7)	53.9mm^3^ ± 40.2 (5.2–111.1mm^3^)	73.8mm^3^ ± 50.7 (3.3–134.4mm^3^)	Not applicable
Diameter (mm)	Total nodules (n = 115)	Total nodules (n = 115)	7.7mm ± 3.5 (3.8–19.4mm)	8.1mm ± 4.0 (3.5–22.8mm)	14 (12.2%)
		Solid nodules (n = 93)	6.4mm ± 1.9 (3.8–12.3mm)	6.6mm ± 2.2 (3.5–12.4mm)	6 (6.5%)
		Part-solid nodules (n = 22)	13mm ± 3.4 (7.2–19.4mm)	14.5mm ± 4.0 (8.6–22.8mm)	8 (36.4%)
		Solid component of part-solid nodules (n = 22)	5.5mm ± 2.9 (1.7–11.9mm)	6.7mm ± 3.2 (1.4–14.3mm)	Not applicable
	Cancer nodule (n = 26)	Total cancer nodules (n = 26)	11.2mm ± 4.3 (4.1–19.4mm)	13.0mm ± 4.3 (7.4–22.8mm)	11 (42.3%)
		Solid nodules (n = 11)	7.9mm ± 2.0 (4.1–10.4mm)	10.1mm ± 1.8 (7.4–12.4mm)	6 (54.5%)
		Part-solid nodules (n = 15)	13.6mm ± 3.9 (7.2–19.4mm)	15.1mm ± 4.3 (8.6–22.8mm)	5 (33.3%)
		Solid component of part-solid nodules (n = 15)	6.0mm ± 3.1 (1.7–11.9mm)	7.2mm ± 3.3 (2.2–14.3mm)	Not applicable
	Benign nodule (n = 89)	Total benign nodules (n = 89)	6.6mm ± 2.4 (3.8–14.1mm)	6.7mm ± 2.7 (3.5–16.0mm)	3 (3.4%)
		Solid nodules (n = 82)	6.2mm ± 1.8 (3.8–12.3mm)	6.2mm ± 1.8 (3.5–10.9mm)	0 (0%)
		Part-solid nodules (n = 7)	11.8mm ± 1.9 (9.6–14.1mm)	13.1mm ± 3.0 (8.8–16.0mm)	3 (42.9%)
		Solid components of part-solid nodules (n = 7)	4.4mm ± 1.7 (1.7–8.0mm)	5.5mm ± 2.7 (1.4–10.5mm)	Not applicable

LDCT: low-dose chest CT

Numbers in parentheses are ranges of average diameter or volume

* Nodule growth between baseline and follow-up LDCTs were adjudicated as per the percentage volume growth of 25% or more (volumetry criterion) and absolute diameter growth more than 1.5 mm (diametric criterion)

### Nodule growth and lung cancer diagnosis

**Fig 3:**
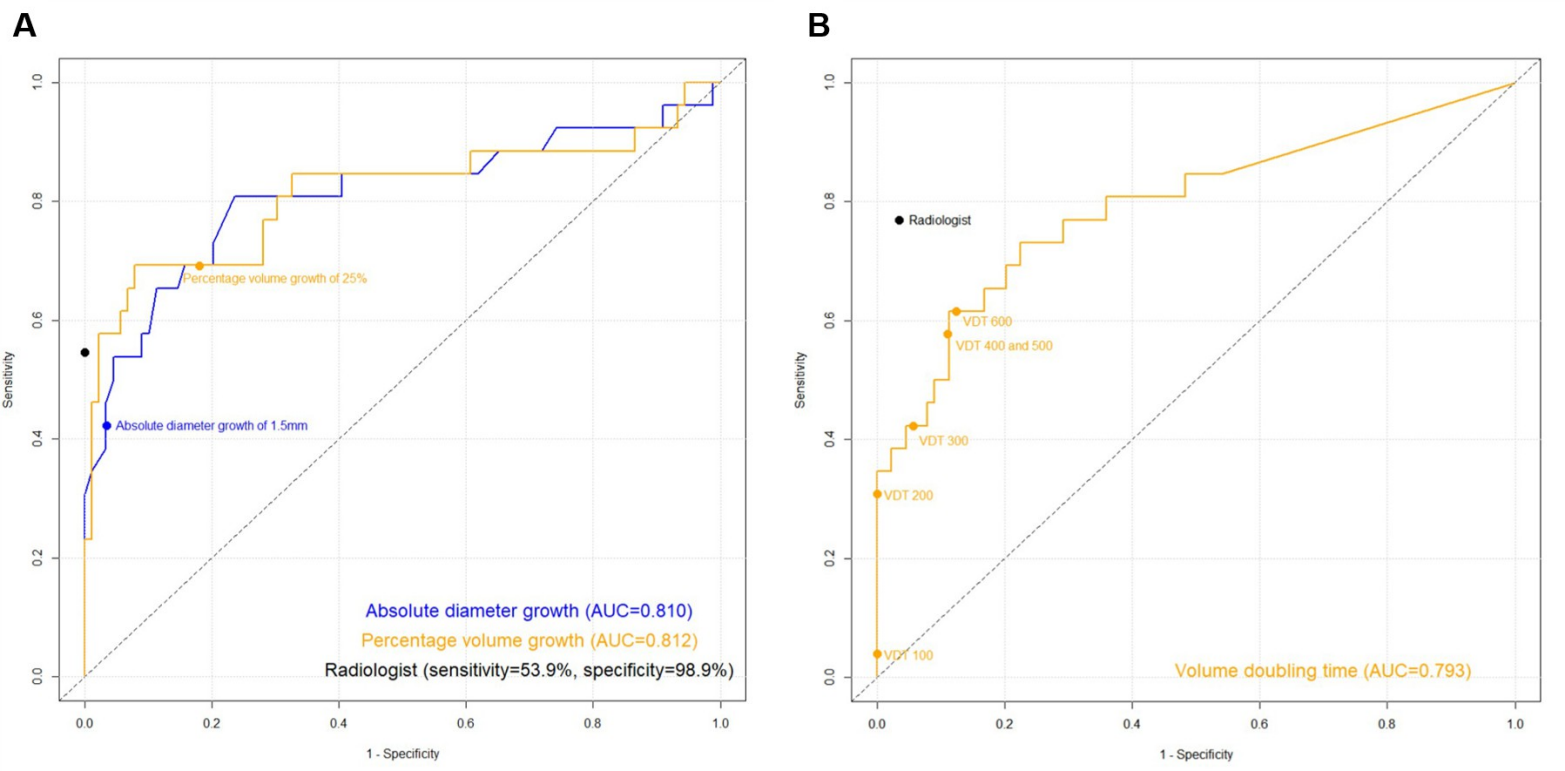
(A) Receiver operating characteristic (ROC) curves of the volumetric and diametric measurement for diagnosing lung cancers. The area under the curve (AUC) values of growth adjudicated by volumetric and diametric measurement for diagnosing lung cancers were 0.812 and 0.810, respectively (*p* = 0.995). (B) ROC curve of volume doubling time for diagnosing lung cancer (AUC, 0.793). The sensitivity and specificity of the radiologist’s diagnostic referral were 76.9% and 96.6%, respectively.

The percentage volume growth and absolute diameter growth based on semi-automated nodule measurements are summarized in Table 1. For the diagnosis of lung cancer, the percentage volume growth and absolute diameter growth exhibited similar AUCs (0.812 [95% CI: 0.694–0.931] vs. 0.810 [95% CI: 0.693–0.927]; *p* = 0.995; Fig 3). At the pre-defined thresholds for growth, percentage volume growth ≥25% exhibited higher sensitivity than absolute diameter growth >1.5 mm (69.2% [95% CI: 51.5–87.0%] vs. 42.3% [95% CI: 23.3–61.3%]; *p* = 0.023). The sensitivity of the subjective evaluation of growth by a radiologist (53.9% [95% CI: 34.7–73.0%]) did not significantly differ between the two pre-defined thresholds. Meanwhile, percentage volume growth ≥25% (82.0% [95% CI: 74.0–90.0%]) exhibited lower specificity than absolute diameter growth >1.5 mm (96.6% [95% CI: 92.9–100%]; *p* = 0.002) and subjective evaluation of growth by radiologist (98.9% (95% CI: 96.7–100%); *p*<0.001) (Table 2).

**Table 2:** Comparison of diagnostic performance for lung cancer diagnosis between growths adjudication of volumetric and diametric measurements, and subjective radiologist’s assessment.

Diagnostic measures		*p*-value	
Criteria	Sensitivity	vs. diametric*	vs. radiologist
Volumetric*	69.2% (51.5–87.0%) [18 of 26]	0.023	0.289
Diametric*	42.3% (23.3–61.3%) [11 of 26]	N.A.	0.505
Radiologist	53.9% (34.7–73.0%) [14 of 26]	0.505	N.A.
Criteria	Specificity	vs. diametric*	vs. radiologist
Volumetric*	82.0% (74.0–90.0%) [73 of 89]	0.002	<0.001
Diametric*	96.6% (92.9–100%) [86 of 89]	N.A.	0.480
Radiologist	98.9% (96.7–100%) [88 of 89]	0.480	N.A.

The numbers in parentheses are 95% confidence intervals. The numbers in brackets are raw data.

* Nodule growth between baseline and follow-up LDCTs were adjudicated as per the percentage volume growth of 25% or more (volumetry criterion) and absolute diameter growth more than 1.5 mm (diametric criterion)

In the subgroup analysis with solid nodules only, percentage volume growth and absolute diameter growth exhibited similar AUCs (0.921 [95% CI: 0.817–1.000] vs. 0.911 [95% CI: 0.806–1.000]; *p* = 0.548; S1 Fig). Percentage volume growth ≥25% (81.8% [95% CI: 59.0–100%]) exhibited slightly higher sensitivity without statistical significance compared to absolute diameter growth >1.5 mm (54.6% [95% CI: 25.1–84.0%]; *p* = 0.083) and subjective evaluation of growth by radiologist (54.6% [95% CI: 25.1–84.0%]; *p* = 0.083). Meanwhile, percentage volume growth ≥25% (84.2% [95% CI: 76.2–92.1%]) exhibited lower specificity than absolute diameter growth >1.5 mm (100% [95% CI: 95.6–100%]; *p*<0.001) and subjective evaluation of growth by radiologist (100% [95% CI: 95.6–100%]; *p*<0.001) (S1 Table).

### Volume doubling time and lung cancer diagnosis

Among the 25 nodules that exhibited volume growth ≥25%, average VDT ± standard deviation was 260.3 days ± 100.0 (range, 70.8–391.3 days). Solid nodules (n = 13), ground-glass and solid component of part-solid nodules (n = 12) exhibited average VDT of 275.5 days ± 95.4 (range, 118.0–387.7 days), 243.8 days ± 106.3 (range, 70.8–391.3 days), and 243.8 days ± 106.4 (range, 70.8–391.3 days), respectively. For the diagnosis of lung cancer, the VDT exhibited an AUC of 0.793 (95% CI: 0.707–0.863) (Fig 3). The sensitivities and specificities of the different VDT thresholds are described in Table 3 and S2 Fig, respectively. VDT thresholds of 200 days (sensitivity, 30.8% [95% CI: 13.0–48.5%]; specificity, 100% [95% CI: 95.9–100%]) exhibited significantly lower sensitivity than VDT threshold of 600 days (61.5% [95% CI: 42.8–80.2%]; specificity, 87.6% [95% CI: 80.8–94.5%]), while VDT thresholds of 300 days or shorter exhibited significantly higher specificity than VDT threshold of 600 days.

**Table 3:** Diagnostic performance of volume doubling time for lung cancer diagnosis in 115 indeterminate nodules detected in baseline screening CT.

Threshold of VDT	Sensitivity	*p*-value (vs. VDT of 600)	*p*-value (vs. radiologist)	Specificity	*p*-value (vs. VDT of 600)	*p*-value (vs. radiologist)
600 days	61.5% (42.8–80.2%) [16 of 26]	Reference	0.221	87.6% (80.8–94.5%) [78 of 89]	Reference	0.043
500 days	57.7% (38.7–76.7%) [15 of 26]	>0.999	0.131	92.1% (86.5–97.7%) [82 of 89]	0.134	0.289
400 days	57.7% (38.7–76.7%) [15 of 26]	>0.999	0.131	92.1% (86.5–97.7%) [82 of 89]	0.134	0.289
300 days	42.3% (23.3–61.3%) [11 of 26]	0.074	0.016	94.4% (89.6–99.2%) [84 of 89]	0.041	0.683
200 days	30.8% (13.0–48.5%) [8 of 26]	0.013	0.003	100% (95.9–100%) [89 of 89]	0.003	0.248
100 days	3.9% (0–11.2%) [1 of 26]	<0.001	<0.001	100% (95.9–100%) [89 of 89]	0.003	0.248
Diagnostic referral by radiologist	76.9% (60.7–93.1%) [20 of 26]	0.102	Reference	96.6% (92.9–100%) [86 of 89]	0.035	Reference

VDT: volume doubling time

The numbers in parentheses are 95% confidence intervals. The numbers in brackets are raw data.

In the subgroup analysis with solid nodules only, the AUC of VDT for lung cancer diagnosis was 0.867 (95% CI: 0.725–1.000) (S1 Fig). The sensitivities and specificities of the different VDT thresholds are described in S2 Table.

The added value of diagnostic referral by subjective interpretation of the radiologist in all nodules and solid nodules are described in S3 Table and S2 Fig, and S4 Table and S3 Fig, respectively.

### Inter-reader agreement

**Fig 4:**
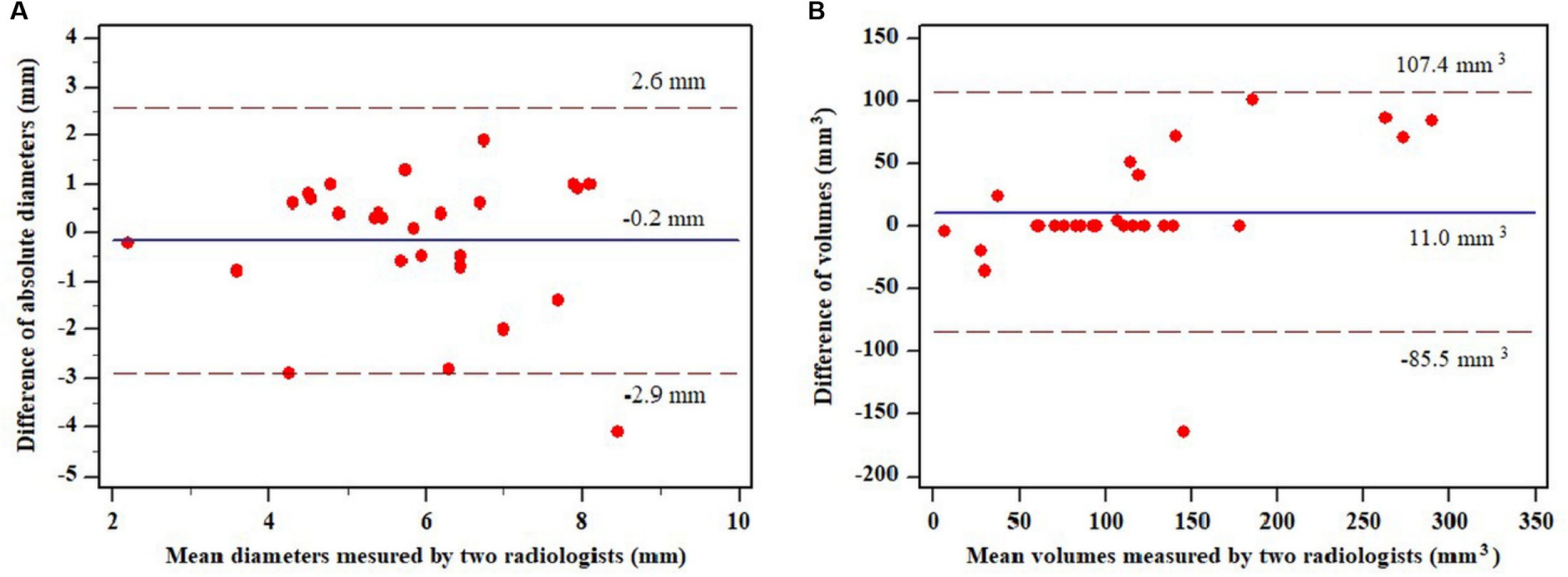
Bland-Altman plots for agreement (A) between mean diameters measured by two radiologists and difference of diameters between the two radiologists (the 95% limit of agreement was between -2.9mm to 2.6mm), and (B) between mean volumes measured by two radiologists and difference of volumes between the two radiologists (the 95% limit of agreement was between -85.5mm^3^ and 107.4mm^3^).

Regarding inter-reader agreement for semi-automated measurement of pulmonary nodules (n = 14), the average diameter and volume of the nodules showed inter-class correlation coefficients of 0.773 (95% CI: 0.509–0.895) and 0.878 (95% CI: 0.736–0.944), respectively. In Bland-Altman plots (Fig 4), 95% limit of agreement for the average diameter of nodules was -2.9–2.6 mm, while that for nodule volume was -85.5 to 107.4 mm^3^. For growth evaluation of these 14 nodules, Cohen’s kappa coefficient and percentage agreement were 0.696 (95% CI: 0.324–1.000) and 85.7% for percentage volume growth ≥25%, 0.054 (95% CI: -0.477–0.585) and 64.3% for absolute diameter growth >1.5 mm, and 0.276 (95% CI: -0.026–0.578) and 57.1% for subjective radiologists’ adjudication, respectively.

Regarding inter-reader agreement of the radiologists’ subjective assessment for nodule growth (n = 115), Cohen’s kappa coefficient and percentage agreement were 0.587 (95% CI: 0.377–0.796) and 89.6%, respectively. Meanwhile, for inter-reader agreement of radiologists’ decision for diagnostic referral, Cohen’s kappa coefficient and percentage agreement were 0.796 (95% CI: 0.661–0.931) and 93.0%, respectively.

## Discussion

In our study, growth of screening-detected indeterminate pulmonary nodules defined as percentage volume growth ≥25% exhibited higher sensitivity and lower specificity for the diagnosis of lung cancer compared to the growth defined as absolute diameter growth >1.5 mm (sensitivity, 69.2% vs. 42.3%; specificity, 82.0% vs. 96.6%). Regarding diagnostic referral based on VDT, thresholds ≤200 and ≤300 days exhibited significantly lower sensitivity (30.8%) and higher specificity (94.4%) than those with a VDT threshold of 600 days (sensitivity, 61.5%; specificity, 87.6%), respectively.

The major advantage of volumetric measurement of pulmonary nodules is that they can sensitively detect nodule growth [12, 14]. The 25% threshold has been considered the margin of measurement variability [23, 24] and has been adopted in several European lung cancer screening trials [4, 25, 26]. The diametric changes of up to 1.5 or 2 mm are usually regarded as measurement variability [10, 11, 13]. Concordant with this, our results suggest that volumetric measurement of lung nodules with a threshold of percentage volume growth ≥25% can detect early lung cancer more sensitively compared to diametrical measurement (69.2% vs. 42.3%), although their AUCs were not significantly different (0.812 vs. 0.810). However, the percentage volume growth ≥25% showed lower specificity than absolute diameter growth >1.5 mm (82.0% vs. 96.6%) in our study. In other words, nodule growth defined by volumetry can lead to false-positive detection of growth in benign nodules, which can be due to measurement variability or true growth of benign nodules.

To reduce false-positive nodule growth, further evaluation of the growth rate of nodules is required. Previous studies have reported a relatively wide range of VDTs for lung cancers, ranging from 100–600 days [11]. Indeed, considering the VDT of lung cancers, previous lung cancer screening trials have adopted a VDT threshold of 400 days for diagnostic referral [4, 27], while the European position statement suggested a more conservative threshold of 600 days [19]. In our study, the 600-day VDT threshold exhibited sensitivity and specificity of 61.5% and 87.6%, respectively, and VDT thresholds <600 days resulted in lower sensitivity and higher specificity. All VDT thresholds showed lower sensitivities than diagnostic referrals based on the subjective decision of the radiologist, suggesting that a substantial proportion of lung cancer patients might undergo diagnostic delays in VDT-based diagnostic referral only. This could be due to pulmonary adenocarcinomas appearing as part-solid nodules, which usually show relatively longer VDTs [11]. Indeed, in our study, 17 of 26 lung cancers (65.4%; solid nodules, n = 8; part-solid nodules, n = 9) had VDTs ranging from 100–600 days, the other 9 lung cancers (34.6%) had VDTs shorter than 100 days (part-solid nodule, n = 1) or longer than 600 days (solid, n = 3; part-solid nodules, n = 5). Reflecting this, in a subgroup analysis with only solid nodules, a 600-day VDT threshold exhibited slightly higher sensitivity than the subjective decision by the radiologist (72.7% vs. 63.6%).

It would be difficult to define an optimum VDT threshold because there is a trade-off between the benefit of sensitive detection of early lung cancer and the cost of unnecessary diagnostic referral or invasive procedures. A previous study by Heuvelmans *et al*. suggested a 232-day VDT threshold for the identification of lung cancers in three-month follow-up LDCTs [28]. However, in our study, VDT thresholds ≤200 days led to a substantial reduction in sensitivity. Nonetheless, adding subjective decisions for diagnostic referral by a radiologist can improve the balance between sensitivity and specificity. In our study, the VDT threshold of 200 days with combined radiologist adjudication for diagnostic referral resulted in the same sensitivity at higher specificity compared with the VDT threshold of 600 days.

Reduced inter- or intra-reader variability is another key advantage of volumetric lung nodule measurements using segmentation. Simple diametric measurement or volume measurement by simply using tumor diameter cannot reflect the three-dimensional nature of pulmonary nodules and therefore are prone to inter- or intra-reader variability [10, 12, 29]. Concordant with these previous studies [10, 12, 14, 29, 30], our results corroborated a higher kappa coefficient value (0.696) and percentage agreement (85.7%) for nodule growth with volumetric measurement than those of diametric measurement (kappa coefficient = 0.054, percentage agreement = 64.3%) and subjective radiologists’ adjudication (kappa coefficient = 0.276, percentage agreement = 57.1%).

This study had several limitations. First, the retrospective nature and relatively small study population might have limited our study’s results. For example, heterogeneity of CT scanners or protocols, even the baseline and follow-up CT in one individual, could affect the result, but this was inevitable due to the retrospective nature of this study. To overcome this limitation, a prospective study with uniform CT scanners and protocols should be warranted. Alternatively, applying state-of-the-art techniques such as the deep learning-based image reconstruction kernel conversion model can be helpful. Second, a diagnostic case-control study, in which researchers collect disease-positive and disease-negative cases through convenience sampling, cannot reflect real-world screening settings from the perspective of unrealistic disease prevalence. Indeed, this study has a selection bias in that two cohorts with different characteristics were included. That is, while individuals in the K-LUCAS project were included regardless of whether lung cancer was diagnosed, individuals in our institution were included if they were diagnosed with lung cancer. Third, although inter-observer variabilities were investigated in measuring average diameter and volume, and subjective assessment, only two radiologists participating in this study may limit the generalizability of the results. Furthermore, because various types of commercially available segmentation software can affect the measurement and classification of nodules [12], we used only a single software.

In conclusion, growth evaluation of screening-detected indeterminate nodules with volumetric measurement exhibited higher sensitivity but lower specificity compared to diametric measurements.

## Supporting information

S1 Table Comparison of diagnostic performance for lung cancer diagnosis between growth adjudication of volumetric and diametric measurements and subjective radiologist’s assessment in 93 solid nodules. (DOCX)

S2 Table Diagnostic performance of volume doubling time for lung cancer diagnosis in 93 indeterminate solid nodules detected in baseline screening CT. (DOCX)

S3 Table The added value of diagnostic referral by subjective interpretation of radiologist to volume doubling time for lung cancer diagnosis. (DOCX)

S4 Table The added value of diagnostic referral by subjective interpretation of radiologist to volume doubling time for lung cancer diagnosis in 93 solid nodules. (DOCX)

S1 Fig Subgroup analyses with solid nodules in the baseline screening CT. (A) Receiver operating characteristic (ROC) curves of the volumetric and diametric measurement for diagnosing lung cancers. The area under the curve (AUC) values of growth adjudicated by volumetric and diametric measurement for diagnosing lung cancers were 0.921 and 0.911, respectively (*p* = 0.477). (B) ROC curve of volume doubling time for diagnosing lung cancer (AUC, 0.867). The sensitivity and specificity of the radiologist’s diagnostic referral were 63.6% and 100%, respectively.
(TIF)

S2 Fig (A) Sensitivities of volumetric measurement for diagnosing lung cancers with or without diagnostic referral by the radiologist. (B) Specificity of volumetric measurement for diagnosing lung cancers with or without diagnostic referral by the radiologist. With the subjective assessment of the radiologist, sensitivities improved with maintained specificities in volume doubling time thresholds of 500 days or shorter.
(TIF)

S3 Fig Subgroup analyses with solid nodules in the baseline screening CT. (A) Sensitivities of volumetric measurement for diagnosing lung cancers with or without diagnostic referral by the radiologist. (B) Specificity of volumetric measurement for diagnosing lung cancers with or without diagnostic referral by the radiologist (overlapped on the plot). With the subjective assessment of the radiologist, sensitivities improved with maintained specificities in volume doubling time thresholds of 300 days or shorter.
(TIF)

S1 Dataset Minimal data set for this study. (XLSX)

## Abbreviations

AUC: area under the receiver operating characteristic curve
LDCT: low-dose chest CT
VDT: volume doubling time
NELSON: Dutch-Belgian Lung Cancer Screening Trial
