# Supplementary material for: Determination of the optimum definition of growth evaluation for indeterminate pulmonary nodules detected in lung cancer screening
Source: PLoS One. 2022 Sep 15;17(9):e0274583. doi: 10.1371/journal.pone.0274583 (PMC9477274; doi:10.1371/journal.pone.0274583)
Supplement: S1 Table — (DOCX) [file pone.0274583.s001.docx]

**S1 Table. Comparison of diagnostic performance for lung cancer diagnosis between growth adjudication of volumetric and diametric measurements and subjective radiologist’s assessment in 93 solid nodules.**

| Diagnostic measures | | *p*-value | |  |
| --- | --- | --- | --- | --- |
|  | Sensitivity | vs. diametric* | vs. radiologist | |
| Volumetric* | 81.8% (59.0–100%)  [9 of 11] | 0.083 | 0.083 | |
| Diametric* | 54.6% (25.1–84.0%)  [6 of 11] | N.A. | >0.999 | |
| Radiologist | 54.6% (25.1–84.0%)  [6 of 11] | >0.999 | N.A. | |
|  | Specificity | vs. diametric* | vs. radiologist | |
| Volumetric* | 84.2% (76.2–92.1%)  [69 of 82] | <0.001 | <0.001 | |
| Diametric* | 100% (95.6–100%)  [82 of 82] | N.A. | >0.999 | |
| Radiologist | 100% (95.6–100%)  [82 of 82] | >0.999 | N.A. | |

The numbers in parentheses are 95% confidence intervals. The numbers in brackets are raw data.

* Nodule growth between baseline and follow-up LDCTs were adjudicated as per the percentage volume growth of 25% or more (volumetry criterion) and absolute diameter growth of more than 1.5 mm (diametric criterion)
