# Supplementary material for: Determination of the optimum definition of growth evaluation for indeterminate pulmonary nodules detected in lung cancer screening
Source: PLoS One. 2022 Sep 15;17(9):e0274583. doi: 10.1371/journal.pone.0274583 (PMC9477274; doi:10.1371/journal.pone.0274583)
Supplement: S2 Table — (DOCX) [file pone.0274583.s002.docx]

**S2 Table. Diagnostic performance of volume doubling time for lung cancer diagnosis in 93 indeterminate solid nodules detected in baseline screening CT**

| VDTs | Sensitivity | *p*-value (vs. VDT of 600) | *p*-value (vs. radiologist) | Specificity | *p*-value (vs. VDT of 600) | *p*-value (vs. radiologist) |
| --- | --- | --- | --- | --- | --- | --- |
| 600 days | 72.7% (46.4–99.1%)  [8 of 11] | Reference | >0.999 | 90.2% (83.8–96.7%)  [74 of 82] | Reference | 0.013 |
| 500 days | 63.6% (35.2–92.1%)  [7 of 11] | >0.999 | >0.999 | 93.9% (88.7–99.1%)  [77 of 82] | 0.248 | 0.074 |
| 400 days | 63.6% (35.2–92.1%)  [7 of 11] | >0.999 | >0.999 | 93.9% (88.7–99.1%)  [77 of 82] | 0.248 | 0.074 |
| 300 days | 45.5% (16.0–74.9%)  [5 of 11] | 0.248 | 0.617 | 96.3% (92.3–100%)  [79 of 82] | 0.074 | 0.248 |
| 200 days | 36.4% (7.9–64.8%)  [4 of 11] | 0.134 | 0.371 | 100% (95.6–100%)  [82 of 82] | 0.013 | N.A. |
| 100 days | 0% (0–28.5%)  [0 of 11] | 0.013 | 0.023 | 100% (95.6–100%)  [82 of 82] | 0.013 | N.A. |
| Diagnostic referral by radiologist | 63.6% (35.2–92.1%)  [7 of 11] | 0.317 | Reference | 100% (95.6–100%)  [82 of 82] | 0.008 | Reference |

VDT: volume doubling time

The numbers in parentheses are 95% confidence intervals. The numbers in brackets are raw data.
