# Supplementary material for: Determination of the optimum definition of growth evaluation for indeterminate pulmonary nodules detected in lung cancer screening
Source: PLoS One. 2022 Sep 15;17(9):e0274583. doi: 10.1371/journal.pone.0274583 (PMC9477274; doi:10.1371/journal.pone.0274583)
Supplement: S3 Table — (DOCX) [file pone.0274583.s003.docx]

**S3 Table. The added value of diagnostic referral by subjective interpretation of radiologist to volume doubling time for lung cancer diagnosis**

|  | Sensitivity | *p*-value (vs. each VDT) | Specificity | *p*-value (vs. each VDT) |
| --- | --- | --- | --- | --- |
| Diagnostic referral by radiologist | 76.9% (60.7–93.1%)  [20 of 26] | N.A. | 96.6% (92.9–100%)  [86 of 89] | N.A. |
| VDT 600 days | 61.5% (42.8–80.2%)  [16 of 26] | Reference | 87.6% (80.8–94.5%)  [78 of 89] * | Reference |
| VDT 600 days + radiologist | 80.8% (65.6–95.9%)  [21 of 26] | 0.074 | 84.3% (76.7–91.8%)  [76 of 89] | 0.480 |
| VDT 500 days | 57.7% (38.7–76.7%)  (15 of 26) | Reference | 92.1% (86.5–97.7%)  [82 of 89] * | Reference |
| VDT 500 days + radiologist | 80.8% (65.6–95.9%)  [21 of 26] | 0.041 | 89.9% (83.6–96.2%)  [80 of 89] | 0.480 |
| VDT 400 days | 57.7% (38.7–76.7%)  [15 of 26] | Reference | 92.1% (86.5–97.7%)  [82 of 89] * | Reference |
| VDT 400 days + radiologist | 80.8% (65.6–95.9%)  [21 of 26] | 0.041 | 89.9% (83.6–96.2%)  [80 of 89] | 0.480 |
| VDT 300 days | 42.3% (23.3–61.3%)  [11 of 26] * | Reference | 94.4% (89.6–99.2%)  [84 of 89] | Reference |
| VDT 300 days + radiologist | 80.8% (65.6–95.9%)  [21 of 26] | 0.004 | 92.1% (86.5–97.7%)  [82 of 89] | 0.480 |
| VDT 200 days | 30.8% (13.0–48.5%)  [8 of 26] * | Reference | 100% (95.9–100%)  [89 of 89] | Reference |
| VDT 200 days + radiologist | 80.8% (65.6–95.9%)  [21 of 26] | <0.001 | 96.6% (92.9–100%)  [86 of 89] | 0.248 |
| VDT 100 days | 3.9% (0–11.2%)  [1 of 26] * | Reference | 100% (95.9–100%)  [89 of 89] | Reference |
| VDT 100 days + radiologist | 76.9% (60.7–93.1%)  [20 of 26] | <0.001 | 96.6% (92.9–100%)  [86 of 89] | 0.248 |

VDT: volume doubling time

The numbers in parentheses are 95% confidence intervals. The numbers in brackets are raw data.

* *p*<0.05 in comparison with sensitivity or specificity of the radiologist.
