# Supplementary material for: Determination of the optimum definition of growth evaluation for indeterminate pulmonary nodules detected in lung cancer screening
Source: PLoS One. 2022 Sep 15;17(9):e0274583. doi: 10.1371/journal.pone.0274583 (PMC9477274; doi:10.1371/journal.pone.0274583)
Supplement: S4 Table — (DOCX) [file pone.0274583.s004.docx]

**S4 Table. The added value of diagnostic referral by subjective interpretation of radiologist to volume doubling time for lung cancer diagnosis in 93 solid nodules**

|  | Sensitivity | *p*-value (vs. each VDT) | Specificity | *p*-value (vs. each VDT) |
| --- | --- | --- | --- | --- |
| Diagnostic referral by radiologist | 63.6% (35.2 – 92.1%)  [7 of 11] | N.A. | 100% (95.6 – 100%)  [82 of 82] | N.A. |
| VDT 600 days | 72.7% (46.4 – 99.1%)  [8 of 11] | Reference | 90.2% (83.8 – 96.7%)  [74 of 82] * | Reference |
| VDT 600 days + radiologist | 72.7% (46.4 – 99.1%)  [8 of 11] | N.A. | 90.2% (83.8 – 96.7%)  [74 of 82] | N.A. |
| VDT 500 days | 63.6% (35.2 – 92.1%)  [7 of 11] | Reference | 93.9% (88.7 – 99.1%)  [77 of 82] * | Reference |
| VDT 500 days + radiologist | 72.7% (46.4 – 99.1%)  [8 of 11] | 0.317 | 93.9% (88.7 – 99.1%)  [77 of 82] | N.A. |
| VDT 400 days | 63.6% (35.2 – 92.1%)  [7 of 11] | Reference | 93.9% (88.7 – 99.1%)  [77 of 82] * | Reference |
| VDT 400 days + radiologist | 72.7% (46.4 – 99.1%)  [8 of 11] | 0.317 | 93.9% (88.7 – 99.1%)  [77 of 82] | N.A. |
| VDT 300 days | 45.5% (16.0 – 74.9%)  [5 of 11] | Reference | 96.3% (92.3 – 100%)  [79 of 82] | Reference |
| VDT 300 days + radiologist | 72.7% (46.4 – 99.1%)  [8 of 11] | 0.083 | 96.3% (92.3 – 100%)  [79 of 82] | N.A. |
| VDT 200 days | 36.4% (7.9 – 64.8%)  [4 of 11] | Reference | 100% (95.6 – 100%)  [82 of 82] | Reference |
| VDT 200 days + radiologist | 72.7% (46.4 – 99.1%)  [8 of 11] | 0.046 | 100% (95.6 – 100%)  [82 of 82] | N.A. |
| VDT 100 days | 0% (0 – 28.5%)  [0 of 11] * | Reference | 100% (95.6 – 100%)  [82 of 82] | Reference |
| VDT 100 days + radiologist | 63.6% (35.2 – 92.1%)  [7 of 11] | N.A. | 100% (95.6 – 100%)  [82 of 82] | N.A. |

VDT: volume doubling time

The numbers in parentheses are 95% confidence intervals. The numbers in brackets are raw data.

* *p*<0.05 in comparison with sensitivity or specificity of the radiologist.
