# Supplementary figures and images for: Determination of the optimum definition of growth evaluation for indeterminate pulmonary nodules detected in lung cancer screening
Source: PLoS One. 2022 Sep 15;17(9):e0274583. doi: 10.1371/journal.pone.0274583 (PMC9477274; doi:10.1371/journal.pone.0274583)

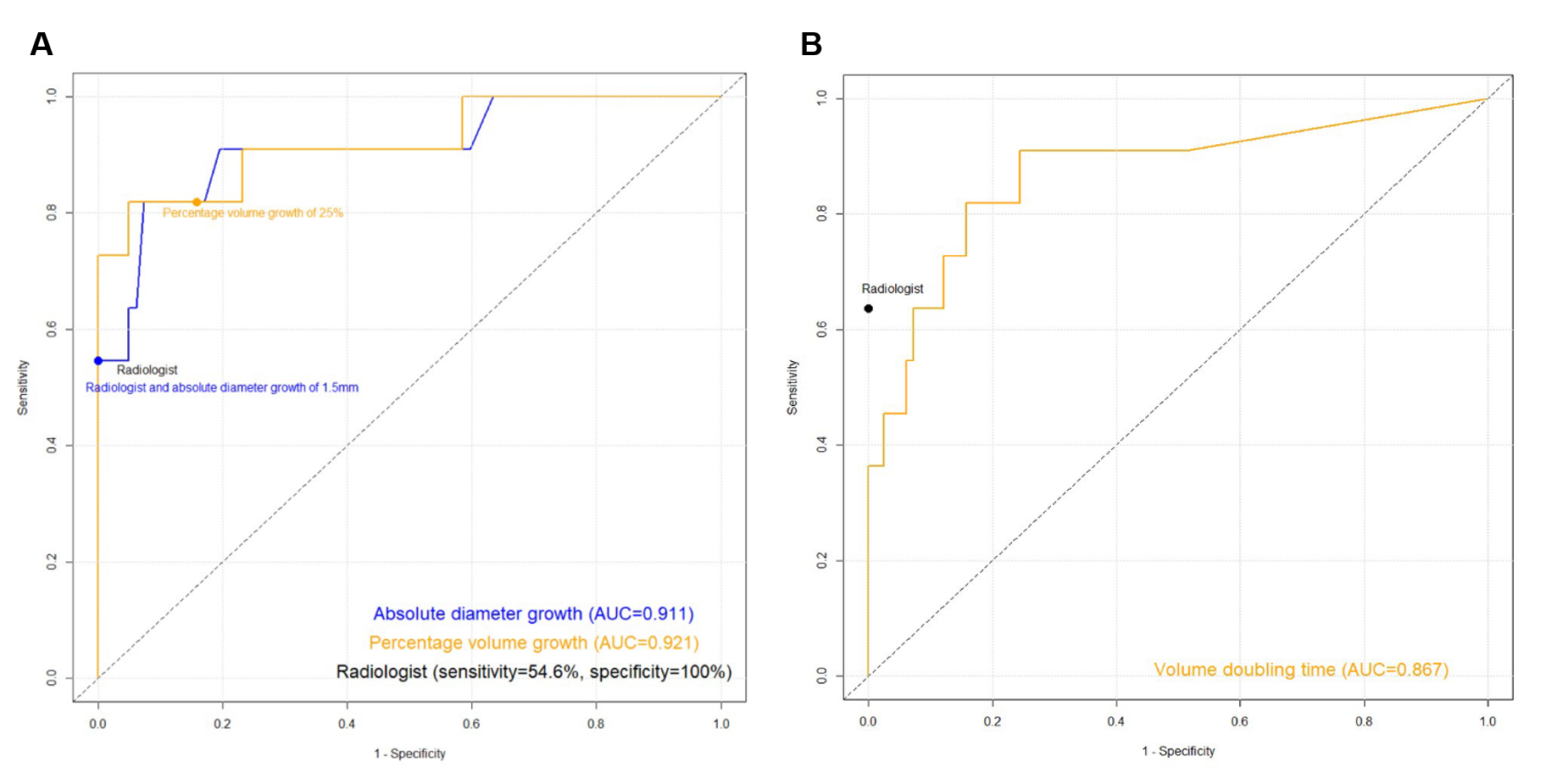

Supplement: S1 Fig — (A) Receiver operating characteristic (ROC) curves of the volumetric and diametric measurement for diagnosing lung cancers. The area under the curve (AUC) values of growth adjudicated by volumetric and diametric measurement for diagnosing lung cancers were 0.921 and 0.911, respectively (p = 0.477). (B) ROC curve of volume doubling time for diagnosing lung cancer (AUC, 0.867). The sensitivity and specificity of the radiologist’s diagnostic referral were 63.6% and 100%, respectively. (TIF) [file pone.0274583.s005.tif]

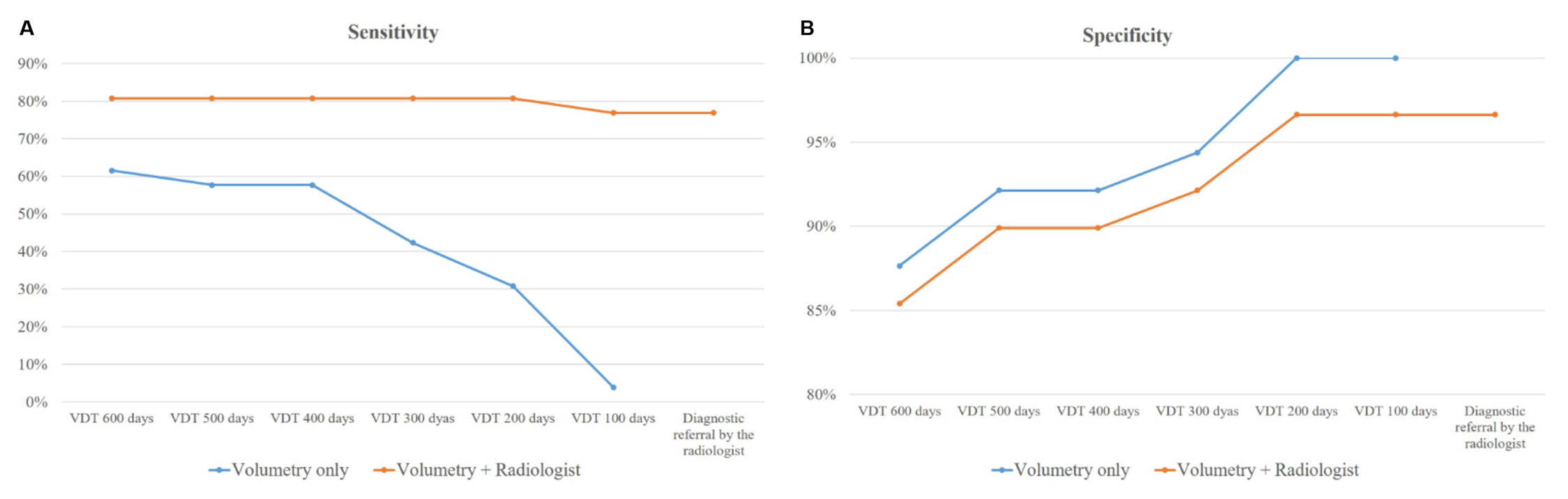

Supplement: S2 Fig — (A) Sensitivities of volumetric measurement for diagnosing lung cancers with or without diagnostic referral by the radiologist. (B) Specificity of volumetric measurement for diagnosing lung cancers with or without diagnostic referral by the radiologist. With the subjective assessment of the radiologist, sensitivities improved with maintained specificities in volume doubling time thresholds of 500 days or shorter. (TIF) [file pone.0274583.s006.tif]

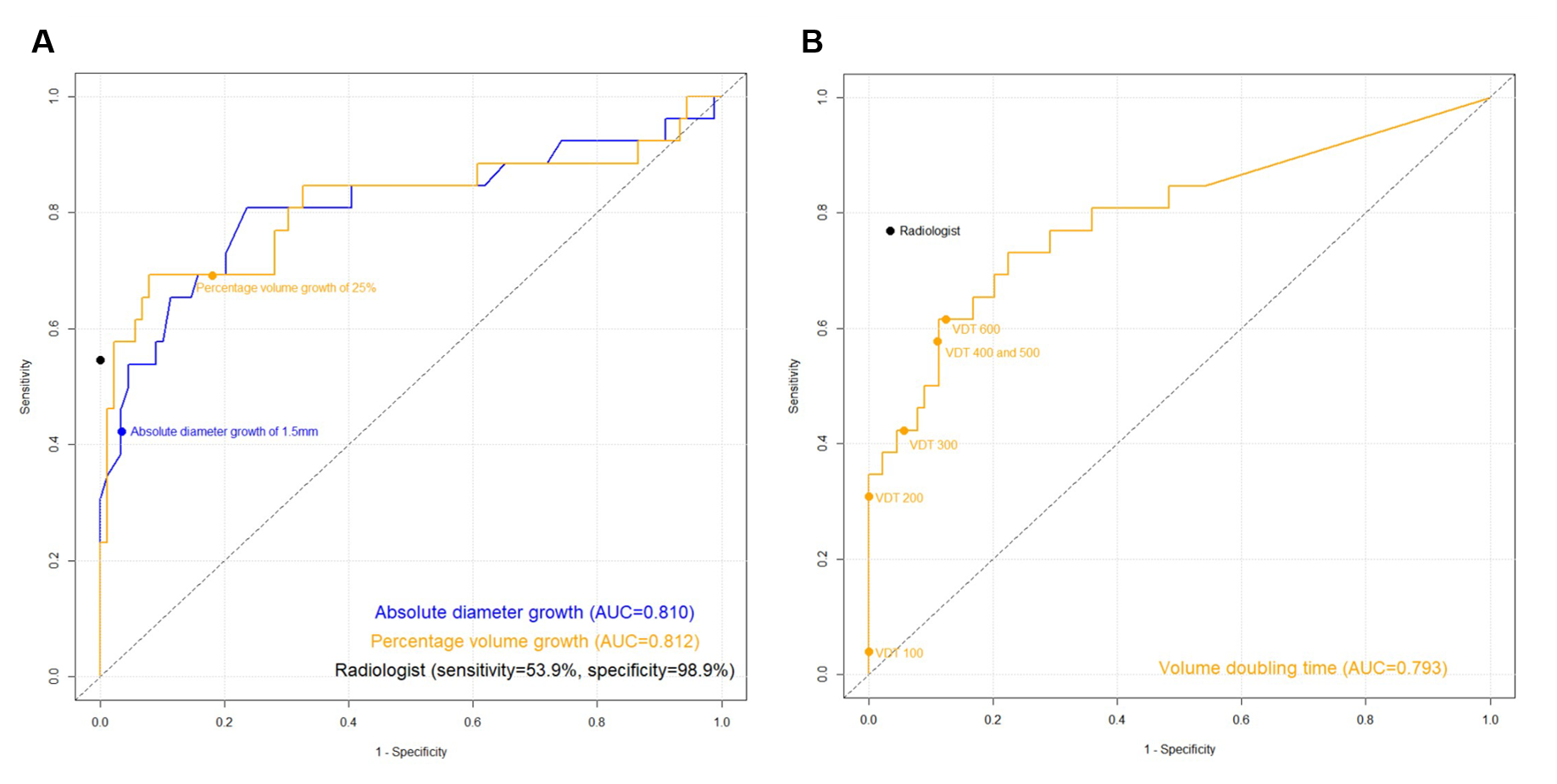

Supplement: S3 Fig — (A) Sensitivities of volumetric measurement for diagnosing lung cancers with or without diagnostic referral by the radiologist. (B) Specificity of volumetric measurement for diagnosing lung cancers with or without diagnostic referral by the radiologist (overlapped on the plot). With the subjective assessment of the radiologist, sensitivities improved with maintained specificities in volume doubling time thresholds of 300 days or shorter. (TIF) [file pone.0274583.s007.tif]
